# Formulation and Evaluation of Moxifloxacin Loaded Bilosomes In-Situ Gel: Optimization to Antibacterial Evaluation

**DOI:** 10.3390/gels8070418

**Published:** 2022-07-04

**Authors:** Ameeduzzafar Zafar, Omar Awad Alsaidan, Syed Sarim Imam, Mohd Yasir, Khalid Saad Alharbi, Mohammad Khalid

**Affiliations:** 1 Department of Pharmaceutics, College of Pharmacy, Jouf University, Sakaka 72341, Al-Jouf, Saudi Arabia; osaidan@ju.edu.sa; 2 Department of Pharmaceutics, College of Pharmacy, King Saud University, Riyadh 11451, Saudi Arabia; simam@ksu.edu.sa; 3 Department of Pharmacy, College of Health Sciences, Arsi University, Asella 396, Ethiopia; mohdyasir@arsiun.edu.et; 4 Department of Pharmacology, College of Pharmacy, Jouf University, Sakaka 72341, Al-Jouf, Saudi Arabia; kssalharbi@ju.edu.sa; 5 Department of Pharmacognosy, College of Pharmacy, Prince Sattam Bin Abdulaziz University, Al-Kharj 11942, Riyadh, Saudi Arabia; m.khalid@psau.edu.sa

**Keywords:** antimicrobial activity, bilosomes, moxifloxacin, ocular delivery, toxicity study

## Abstract

In this study, moxifloxacin (MX)-loaded bilosome (BS) in situ gel was prepared to improve ocular residence time. MX-BSs were prepared using the thin-film hydration method. They were optimized using a Box–Behnken design (BBD) with bile salt (A, sodium deoxycholate), an edge activator (B, Cremophor EL), and a surfactant (C, Span 60) as process variables. Their effects were assessed based on hydrodynamic diameter (Y_1_), entrapment efficacy (Y_2_), and polydispersity index (Y_3_). The optimized formulation (MX-BSop) depicted a low hydrodynamic diameter (192 ± 4 nm) and high entrapment efficiency (76 ± 1%). Further, MX-BSop was successfully transformed into an in situ gel using chitosan and sodium alginate as carriers. The optimized MX-BSop in situ gel (MX-BSop-Ig4) was further evaluated for gelling capacity, clarity, pH, viscosity, in vitro release, bio-adhesiveness, ex vivo permeation, toxicity, and antimicrobial properties. MX-BSop-Ig4 exhibited an optimum viscosity of 65.4 ± 5.3 cps in sol and 287.5 ± 10.5 cps in gel states. The sustained release profile (82 ± 4% in 24 h) was achieved with a Korsmeyer–Peppas kinetic release model (R^2^ = 0.9466). Significant bio-adhesion (967.9 dyne/cm^2^) was achieved in tear film. It also exhibited 1.2-fold and 2.8-fold higher permeation than MX-Ig and a pure MX solution, respectively. It did not show any toxicity to the tested tissue, confirmed by corneal hydration (77.3%), cornea histopathology (no internal changes), and a HET-CAM test (zero score). MX-BSop-Ig4 exhibited a significantly (*p* < 0.05) higher antimicrobial effect than pure MX against *Staphylococcus aureus* and *Escherichia coli*. The findings suggest that bilosome in situ gel is a good alternative to increase corneal residence time, as well as to improve therapeutic activity.

## 1. Introduction

Topical administration (anterior route) is the most common and appropriate route for ophthalmic delivery (eye drops or solution). However, it has low drug bioavailability (about 5–10%) due to short corneal residence time, frequent blinking, high tear-fluid turnover, and elimination via nasolacrimal drainage [1,2]. Due to these drawbacks, a delivery system is needed to avoid frequent administration and to achieve the desired therapeutic activity. Various formulation approaches have been reported for improving bioavailability using viscosity enhancers [3], ointment and gel systems [4], ocular inserts [5], and implant systems [6]. These increase the bioavailability and therapeutic efficacy of the drug, but they have some limitations, such as patient incompliance, difficult administration, and stickiness to the ocular region.

Now, researchers mainly focus on colloidal delivery systems for the improvement of ocular bioavailability and therapeutic efficacy. Different ocular nanocolloidal delivery systems for drugs such as gatifloxacin [7], fluorometholone [8], thymoquinone [9], pilocarpine hydrochloride [10], betaxolol [11], methazolamide [12], dexamethasone [13], ciprofloxacin [14], besifloxacin [15], doxylamine succinate/pyridoxine hydrochloride [16], and clarithromycin [17] have been reported with greater therapeutic activity.

Among them, bilosome (BS)-loaded drug delivery systems are nanosized, ultra-deformable vesicles used for the improvement of ocular delivery [18]. They are composed of a nonionic surfactant, a lipid, and bile salt. They enhance stability, as well as drug encapsulation. Several studies have emphasized the importance of bilosomes as a delivery system [19,20]. The incorporation of bilosomes into an in-situ gel system using a natural biocompatible macromolecular polymer further increases both the ocular residence time and transit time in the cul-de-sac and slows the elimination of drugs through various mechanisms. When administered into the eye, an in-situ gel system converts from a solution to a gel by changing ions, pH, and temperature [21]. Different gel systems, such as calcium iodate particles in a gelatin system and silver nanoparticles, have shown better antimicrobial activity against tested organisms [22,23].

**Figure 1:**
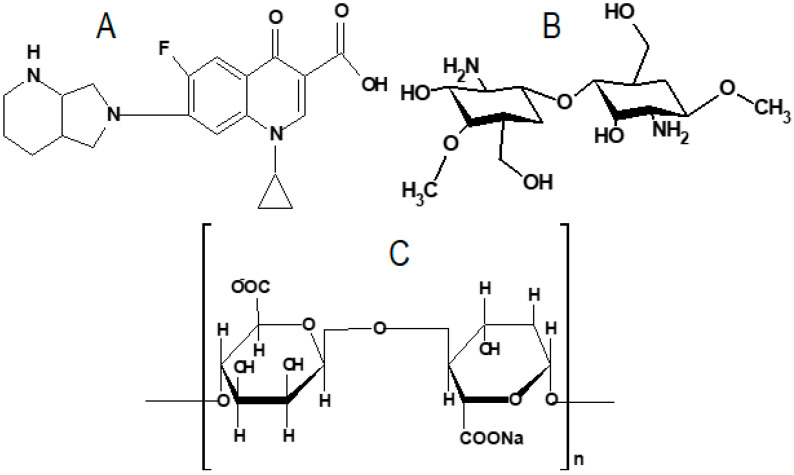
Chemical structures of (**A**) moxifloxacin, (**B**) chitosan, and (**C**) sodium alginate.

Moxifloxacin (MX, Figure 1A) is a broad-spectrum, fourth-generation antibiotic and chemically belongs to the fluoroquinolone derivatives. It is effective against both Gram-positive and Gram-negative bacteria. It kills Gram-negative bacteria by intermingling with DNA gyrase and with topoisomerase IV in Gram-positive bacteria. It is used for the treatment of bacterial conjunctivitis and keratitis [24]. Marketed MX eye drops show that only 5% of the instilled dose reaches the ocular tissue. The remains are eliminated from the eye through an ocular barrier, leading to poor ocular bioavailability [25,26]. MX eye drops (0.5% *w*/*v*, 1 drop, 3 times) were found to be an effective delivery system to treat acute bacterial conjunctivitis [27].

There have been various carrier systems reported by researchers for the improvement of ocular residence time and the therapeutic efficacy of MX. Mudgil et al., formulated an MX-loaded PLGA nanosuspension that exhibited more prolonged in vitro release and higher in vitro activity against *Staphylococcus aureus* (*S. aureus*) and *P. aeruginosa* than pure MX [28]. Albash et al., developed and optimized the terpesomes and leciplex of MX for the enhancement of delivery. The formulation exhibited nanosized particles with high entrapment efficiency and enhancement ratios compared to a pure MX solution. It enhanced the biofilm eradication activity of MX and significantly diminished the bacterial load compared to pure MX [29]. Asfour et al., formulated a chitosan/β-glycerophosphate thermos-sensitive in situ gel of MX for the improvement of therapeutic activity. It exhibited significantly higher antibacterial activity against *S. aureus* than both pure MX and a marketed MX formulation (0.5%) without any damage to the cornea in New Zealand albino rabbits [30]. Magalhaes et al., formulated MX-loaded liposomes and evaluated them in rabbit eyes by injecting them into the anterior chamber. The liposomes demonstrated a smaller size, higher entrapment efficiency, and a more satisfactory release in aqueous humor than pure MX [31]. Sohrabi et al., formulated an MX niosome CS in situ gel and evaluated its physicochemical characterization. The prepared niosome CS in situ gel exhibited higher antibacterial activity against *S. aureus* than niosomes [32]. Darvishi et al., formulated a liposome of MX for pulmonary infection. The liposomes were prepared using a thin-film method and showed a 50–70 nm vesicle size with positive zeta potential. They had a significantly lower minimum inhibitory concentration (MIC) against *P. aeruginosa* and *S. aureus* than pure MX [33].

Chitosan (CH, Figure 1B) is a natural cationic polysaccharide, and it is a key derivative of chitin. It is composed of D-glucosamine and N-acetyl-D glucosamine and is linked with β-1,4 glycosidic linkage. The ratio of these two units determines the degree of acetylation [34]. It is soluble in an acidic environment and generates amino groups (NH^3+^). The positive charge of the CH molecule interacts with the negative group of the microbial membrane and gives antimicrobial activity [35]. It is a pH-sensitive polymer that converts into gel form on contact with simulated tear fluid [36]. Sodium alginate (SA; Figure 1C) is a biopolymer used as a carrier in various dosage forms. It is a salt of alginic acid extracted from brown seaweed. It exhibits immediate gelation by crosslinking with the calcium ions (Ca^2+^) present in tear fluid (pH 7.5) [37]. The use of a combination polymers, i.e., CH (pH) and SA (ion), for the development of in situ gels leads to enhanced corneal residence time, as well as ocular bioavailability [38].

In the present research, an MX-loaded BS in situ gel system is prepared to enhance the ocular residence time. The BSs arere prepared with a thin-film hydration method and are further optimized using a Box–Behnken design based on minimum size and high encapsulation. The optimized BSs are further incorporated into an in-situ gel (Ig) system using biocompatible natural polymers (CH and SA). Finally, the MX-BS-Ig formulation is evaluated for physicochemical characterization, in vitro release, ex vivo permeation, in vitro toxicity, and antimicrobial activity.

## 2. Results and Discussion

### 2.1. Optimization 

**Figure 2:**
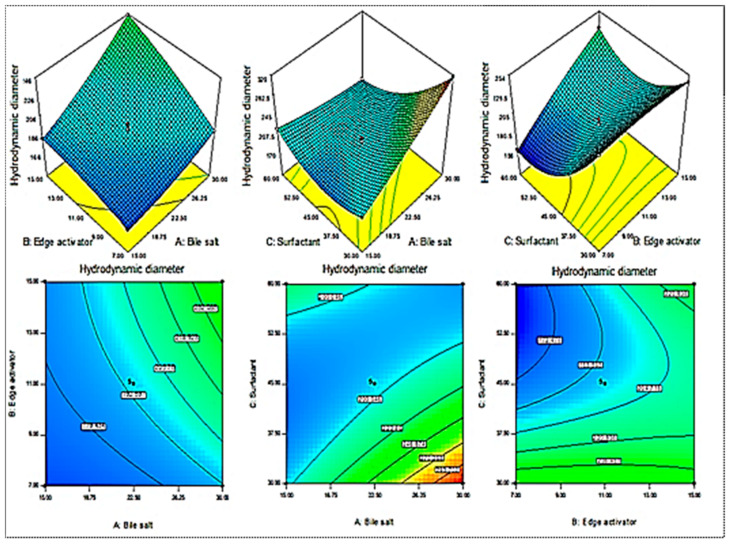
Effect of independent variables sodium deoxycholate (A), Cremophor EL (B), and Span 60 (C) on hydrodynamic diameter (Y_1_).

**Figure 3:**
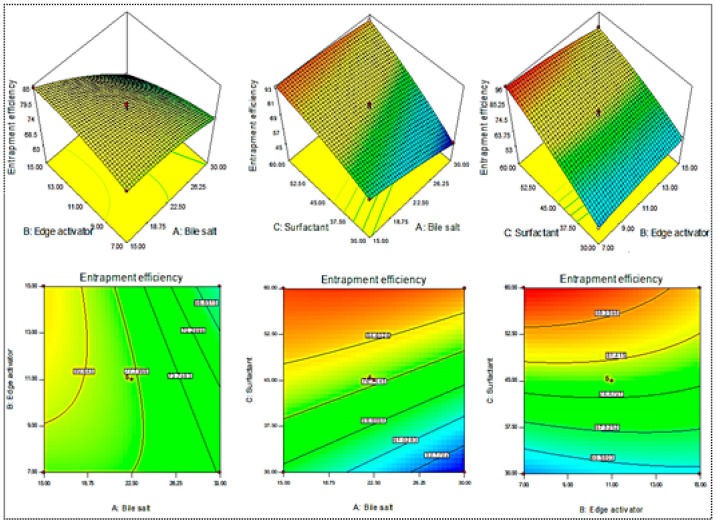
Effect of independent variables (A) sodium deoxycholate, (B) Cremophor EL, and (C) Span 60 on entrapment efficiency (Y_2_).

**Figure 4:**
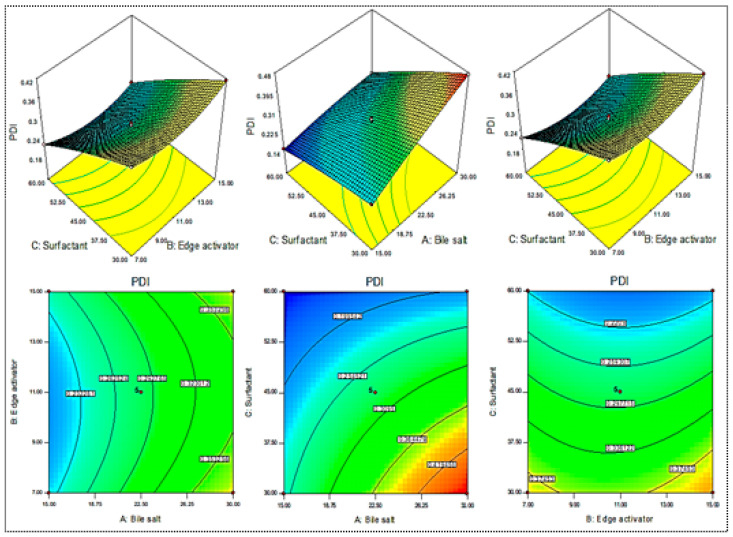
Effect of independent variables sodium deoxycholate (A), Cremophor EL (B), and Span 60 (C) on PDI (Y_3_).

**Figure 5:**
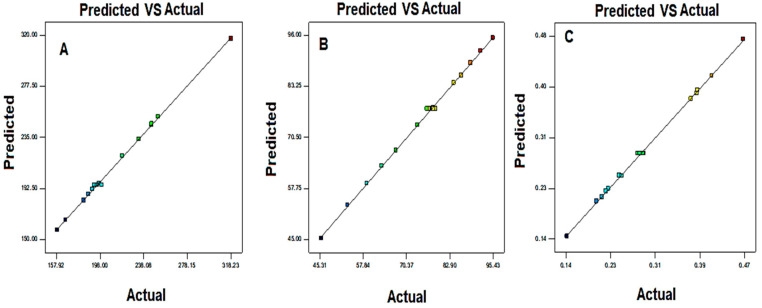
Actual and predicted images of (**A**) hydrodynamic diameter (Y_1_), (**B**) entrapment efficiency (Y_2_), and (**C**) PDI (Y_3_). Color full square indicated the actual and predicted data of the responses.

A Box–Behnken design was used to optimize the MX-BSs by taking different concentrations of bile salt (SDC, A), edge activator (Cremophor EL, B), and surfactant (Span 60, C). Their effects were determined by hydrodynamic diameter (Y_1_), entrapment efficiency (Y_2_), and PDI (Y_3_), as shown in Table 1. The response values were applied to the design space models, i.e., linear, second-order, and quadratic, to assess the effects. The design depicted the quadratic model as the best-fit model for the three variables. The statistical analyses of all the models were analyzed, and the maximum value (R^2^) was found for the quadratic model. An ANOVA of the quadratic model for each response was analyzed, showing it was significantly fitted (*p <* 0.0001) and that the lack of fit was nonsignificant (*p* > 0.05). The 3D response graph (Figure 2, Figure 3 and Figure 4) and the polynomial equations of all the responses showed individual, as well as combined, effects of the independent variables over the responses. The actual and predicted values of all the responses are expressed graphically in Figure 5A–C.

#### 2.1.1. Effects of SDC (A), Cremophor EL (B), and Span 60 (C) on Hydrodynamic Diameter (Y_1_)

The hydrodynamic diameters of the prepared MX-BS formulations were found in the range of 158 nm (MX-BS11) to 318 nm (MX-BS6), as depicted in Table 1. The used variables showed a significant effect on the hydrodynamic diameter, as presented graphically in the 3D response surface plot shown in Figure 2. The formulation of MX-BS11 showed a minimum size with its composition of bile salt (22.5 mg), Cremophor (7 mg), and Span 60 (60 mg). The MS-BS6 formulation depicted the maximum size with bile salt at 30 mg, Cremophor EL at 11 mg, and Span 60 at 30 mg. The significant variations in the results showed that the used variables were important parameters for the optimization process. The increase in the SDC concentration gradually increased the hydrodynamic diameter. The bile salt gave a negative charge to the BSs, and on increasing the concentration of SDC, the negative charge on the BS surface also increased. Due to increased repulsive force in the lipid bilayer, this led to an enhancement in the hydrodynamic diameter [39]. In addition, the hydrodynamic diameter also increased due to the bulkiness characteristic of SDC (heavy structure) [16]. The second factor, Cremophor EL, also showed a significant effect on the hydrodynamic diameter. As the concentration increased, the hydrodynamic diameter also increased due to the formation of a large shield and steric stabilization. The presence of a high number of hydrophilic polyethylene oxide residues (Cremophor EL contains three PEO chains) increased water uptake and led to an increase in size [40,41]. The third factor, Span 60, showed a negative effect, i.e., with the increase in Span 60 concentration, the hydrodynamic diameter decreased due to a reduction in the interfacial tension, which increased the solubility of MX. The quadratic polynomial equation for the hydrodynamic diameter is given below:

Hydrodynamic diameter (Y_1_) = +195.24 + 22.08 A + 17.41 B − 26.71 C + 9.30 AB − 38.09 AC + 20.36 BC + 4.53 A^2^ − 3.31 B^2^ + 30.47 C^2^ (1)

From the equation, it can be observed that variables A, B, and C are coded in terms of bile salt (SDC), edge activator (Cremophor), and surfactant (Span 60), respectively. The terms A, B, and C, as well as AB, AC, BC, A^2^, B^2^, and C^2^ gave significant (*p* < 0.05) effects. These factors showed individual, as well as combined, effects on the hydrodynamic diameter. The positive sign in the equation represents an increase in the effect, whereas the negative sign represents a decrease in the effect. The lack of fit was nonsignificant (F = 0.28, *p* = 0.84), indicating a quadratic model. The close values of the predicted R^2^ (0.9946) and the adjusted R^2^ (0.9969) were found to be in close agreement, as presented graphically in Figure 5A. The adequate precision was >4 (97.08), indicating that the model had an acceptable signal.

#### 2.1.2. Effect of SDC (A), Cremophor EL (B), and Span 60 (C) on Entrapment Efficiency (Y_2_)

The MX-BS6 (45%) formulation, prepared with the composition of 30 mg SDC, 11 mg Cremophor EL, and 30 mg Span 60, had the lowest entrapment efficiency. The maximum entrapment (95%) was found to be for MX-BS11, prepared with a composition of 22.5 mg bile salt, 7 mg Cremophor EL, and 60 mg Span 60, as displayed in Table 1. From the 3D response plot (Figure 3), it was observed that the increase in the bile salt (SDC) concentration led to a reduction in the entrapment efficiency of MX. The decrease in the EE may be due to the formation of micelles in the dispersion medium, which increases solubility. Moreover, with an increase in SDC concentration, the fluidizing effect on the lipid bilayer membrane takes place, resulting in a reduction in entrapment efficiency [42,43]. The second factor, Cremophor EL, showed a negative effect on the entrapment efficiency, but the effect was less than SDC and Span 60. With the increase in the Span 60 concentration, the entrapment efficiency increased. The higher transition temperature, as well as the long alkyl chain of Span 60, helped to obtain a higher EE [20]. The quadratic polynomial equation for entrapment efficiency was constructed using software and is given below:

Entrapment efficiency (Y_2_, %) = +77.63 − 6.25 A − 0.99 B + 17.15 C − 4.09 AB + 4.73 AC − 3.68 BC − 1.46 A^2^ − 1.43 B^2^ − 2.72 C^2^ (2)

From the equation, the factors A, B, C, AB, AC, BC, A^2^, B^2^, and C^2^ were significant model terms (*p* < 0.05) and had significant effects on the entrapment efficiency. These factors showed individual, as well as combined, effects on the EE. The positive sign in the equation was considered for enhancement in the effect, whereas the negative sign was termed for a reduction in the effect. The F-value was 485.44, which suggested that the model was significant (*p* < 0.0001). The lack of fit was nonsignificant (F = 0.18, *p* = 0.90), revealing that the model was well-fitted. The predicted R^2^ (0.9947) was in agreement with the adjusted R^2^ (0.9963), indicating a close relationship between the factors and responses, as shown in Figure 5B. 

#### 2.1.3. Effect of SDC (A), Cremophor EL (B), and Span 60 (C) on PDI (Y_3_)

The PDIs of the prepared MX-BS formulations were found in the range of 0.15 (MX-BS7) to 0.47 (MX-BS6), as depicted in Table 1. The used variables showed significant effects on the PDI, as presented graphically in Figure 4. The formulation of MX-BS7 showed the minimum PDI with a composition of 15 mg bile salt, 11 mg Cremophor, and 60 mg Span 60. The formulation depicting the maximum PDI consisted of 30 mg bile salt, 11 mg Cremophor, and 30 mg Span 60. Significant variation in the results was observed, and it revealed that the used variables were important parameters for the optimization process. The increase in the PDI value was found with the increase in SDC (A). It may be due to an increase in the hydrodynamic diameter of vesicles [44]. The second factor, Cremophor EL (B), showed an insignificant effect on the PDI. However, as the Span 60 (C) concentration increased, the PDI of the MX-BS vesicles decreased due to the reduction in the hydrodynamic diameter. The polynomial equation for PDI was constructed using software and is given below:

PDI (Y_3_) = +0.28 + 0.072 A + 0.003 B − 0.093 C − 0.007 AB − 0.043 AC − 0.008 BC − 0.009 A^2^ + 0.032 B^2^ − 0.007.9 C^2^ (3)

In this equation, A, C, AB, AC, BC, A^2^, B^2^, and C^2^ were significant model terms (*p* < 0.05), and B was an insignificant term (*p* > 0.05). The F-value of 719.23 suggested that the quadratic model was significant (*p* < 0.05). The lack of fit was nonsignificant (*p* > 0.05), and it was good for the model. The predicted R^2^ of 0.992 was in reasonable agreement with the adjusted R^2^ of 0.9975, as depicted in Figure 5C. The adequate precision was found to be >4 (98.57), revealing that the model had an adequate signal.

#### 2.1.4. Selection of Optimized Formulation (MX-BSop)

The selection of the optimized formulation (MX-BSop) was performed using the point prediction method. The selected MX-BSop was prepared with a composition of 18 mg SDC, 13 mg Cremophor EL, and 30 mg Span 60. This composition depicted an experimental hydrodynamic diameter of 192 ± 4 nm, an entrapment efficiency of 76 ± 4%, and a PDI of 0.28 ± 0.03. The software showed a predicted hydrodynamic diameter of 196 nm, an entrapment efficiency of 73%, and a PDI of 0.27. A close agreement was found between the predicted and practical results, revealing that the model was well-fitted, as depicted graphically in Figure 5A–C. The pH of the prepared MX-BS was determined with a digital pH meter and found in the range of 6.1 to 6.2. The drug loading of the MX-BSop formulation was calculated and found to be 10%.

### 2.2. Hydrodynamic Diameter and Zeta Potential

**Figure 6:**
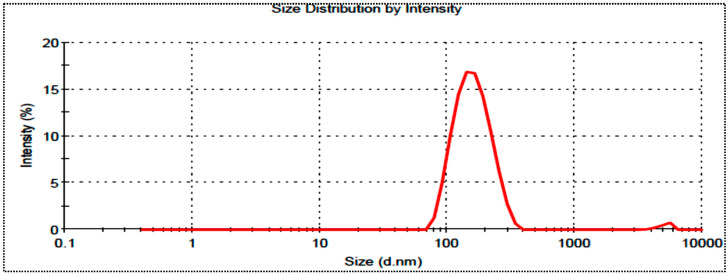
Hydrodynamic diameter image of optimized moxifloxacin bilosomes (MX-BSop).

The hydrodynamic diameters of the prepared MX-BS formulations were found in the range of 158 nm (MX-BS11) to 318 nm (MX-BS6). The point-prediction-based optimized formulation, MX-BSop, showed a hydrodynamic diameter of 192 ± 4 nm (Figure 6) and a PDI of 0.28 ± 0.04. The value of the PDI was found in the standard limit. It showed that the prepared formulation was in a homogenous dispersion. The zeta potential of MX-BSop was found to be −23.5 mV. 

### 2.3. Development of In Situ Gel

The MX-BSop formulation was successfully transformed into an in-situ gel system by using a fixed concentration of CH and varying concentrations of SA as gelling agents. The detailed composition is expressed in Table 2. 

### 2.4. MX-BS-Ig Characterization

The gelling strengths of the MX-BSop-Ig formulations were determined, and the comparative results are given in Table 2. MX-BSop-Ig1 showed no gelation and remained as a liquid (denoted by a negative sign in Table 2). MX-BSop-Ig2 showed slight gelation properties, and the formation of a gel took place within a minute, but it was converted back to a solution form quickly (1 h). MX-BSop-Ig3 and MX-BSop-Ig4 showed higher gelation properties, as denoted by the ++ and +++ signs. The MX-BSop-Ig4 formulation converted to gel form in a few seconds and showed greater stability (>24 h). However, MX-BSop-Ig5 formed a gel quickly, but it was highly viscous (turbid). From the results, it was observed that, at a fixed concentration of CH and increasing concentrations of SA, the gelling capacity increased due to an increase in the viscosity. The mechanism of gel formation took place by the ionic bond interaction between chitosan and sodium alginate [45]. Therefore, the MX-BSop-Ig4 formulation exhibited good gelling strength and was considered as the optimized formulation. The clarity of all the MX-BSop-Ig formulations was determined at the sol state, and the results are presented in Table 2. The samples were observed on a white-and-black background and did not show any foreign particles or precipitation. The pH was almost the same in the sol state, i.e., 6.2 ± 0.02. The percentages of transmittance of all the formulations were measured and found in the range of 80.2 ± 2.3% to 97.3 ± 1.4% (Table 2). Viscosity is very important for topical ocular administration because the formulation is eliminated from the cul-de-sac due to high blinking (17 blinks/min). It has a direct effect on the gelling capacity and stability, as well as on the release of the drugs [45]. The viscosities of the prepared MX-BSop-Ig formulations in sol state (pH 5.5) and gel state (STF, 7.4) were determined, and the results are depicted in Table 2. In the sol state, the viscosity was found in the range of 8.4 ± 1.6 (MX-BSop-Ig1) to 98.5 ± 2.1 cps (MX-BSop-Ig6). The viscosity in the gel state was evaluated in a physiological solution (STF, pH 7.4) and found in the range of 28.4 ± 3.8 (MX-BSop-Ig1) to 314.7 ± 1.1 (MX-BSop-Ig6). It showed that, on increasing the concentration of SA at a fixed concertation of CH, the viscosity of the in-situ gel system increased in both states (sol and gel). The viscosity of the gel system increased due to polymer crosslinking with ions present in the STF, as well as due to stimulation by pH [46]. MX-BSop-Ig4 was selected as the optimized formulation due to the formation of a soft gel on contact with STF, and it remained stable for more than 24 h. The drug content of the MX-BSop-Ig formulations ranged from 97.5 ±2.7% to 99.2 ±2.0%. The optimized in situ gel (MX-BSop-Ig4) depicted a drug content of 99.1 ± 1.2.

#### 2.4.1. DSC Study 

**Figure 7:**
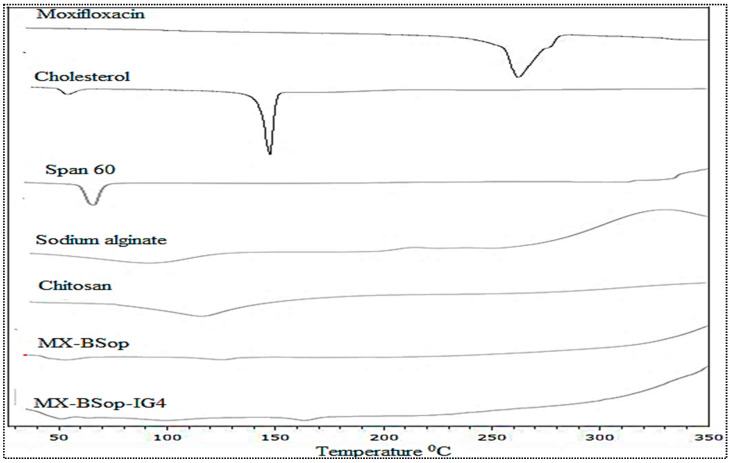
DSC thermogram of moxifloxacin, cholesterol, Span 60, sodium alginate, chitosan, optimized moxifloxacin bilosomes (MX-BSop), and optimized moxifloxacin bilosome in situ gel (MX-BSop-Ig4).

Figure 7 shows a DSC thermogram of MX, CHO, Span 60, SA, CH, MX-BSop, and MX-BSop-Ig4. The pure MX showed a characteristic endothermic peak at 252 °C that was close to the reported melting point and confirmed its purity and crystallinity. The thermograms of CHO, Span 60, SA, and CH exhibited characteristic melting point peaks at 151°C, 68 °C, 89 °C, and 133 °C, respectively. The prepared MX-BSop did not show any characteristic peaks of MX, and only small and less intense peaks for CHO and Span 60 were observed. A similar type of thermogram was also observed for MX-BSop-Ig4, which also did not show a peak for MX. The absence of the MX peak in the MX-BSop and MX-BSop-Ig4 thermogram was due to complete encapsulation in the polymer matrix.

#### 2.4.2. FTIR Spectroscopy Study

The IR spectra of MX, CHO, Span 60, SA, CH, MX-BSop, and MX-BSop-Ig4 were recorded using an FTIR instrument, and the results are depicted in Supplementary Figure S1. The spectrum of MX showed characteristic peaks at 3249 cm^−1^ (O–H stretching vibration of COOH), 3074 cm^−1^ (C–H stretching vibration of aromatic ring), 1714 cm^−1^ (C=O stretching vibration of COOH), 1614 cm^−1^ (C=O phenyl breathing), and 1443 cm^−1^ (C–H deformation), assuring the purity of the MX. The CHO spectrum showed peaks at 3383 cm^−1^ (O–H stretching vibration), 2930 cm^−1^ (CH_2_ symmetric stretching vibration), 2865 cm^−1^ (CH_3_ symmetric stretching vibration), 1442 cm^−1^ (CH_2_ bending), and 1049 cm^−1^ (CH bending). Span 60 showed characteristic peaks at 3301 cm^−1^ (O–H stretching vibration), 2866 cm^−1^ (COOH group stretching), and 1585 cm^−1^ (C–H bending). SA and CH showed characteristic peaks at 880 cm^−1^ (mannuronic acid), 1056 cm^−1^ (uronic acid), 2283 cm^−1^ (OH stretching), and 2928 cm^−1^ (CH_2_ stretching). Chitosan showed a characteristic peak at 3289 cm^−1^ (OH stretching), with C–H peaks (2899 cm^−1^) and CH_2_–OH vibration peaks (1361 cm^−1^). However, in the spectra of MX-BSop and MX-BSop-Ig4, there were non-significant changes in the spectral peaks. The MX and ingredient peaks present revealed an absence of physicochemical interaction between MX and the ingredients.

#### 2.4.3. In Vitro Dissolution Study

**Figure 8:**
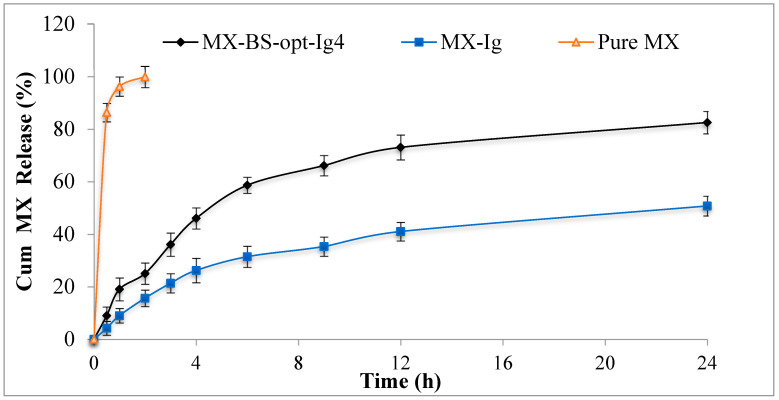
In vitro drug release profiles of MX from moxifloxacin-loaded bilosome in situ gel (MX-BSop-Ig4), moxifloxacin in situ gel (MX-lg), and pure MX solution. The study was performed in triplicate, and data are shown as mean ± SD.

The drug release study of MX-BSop-Ig4, MX-Ig, and the pure MX solution was conducted using a dialysis bag, and the results are depicted in Figure 8. The released MX amounts from MX-BSop-Ig4 and MX-Ig were found to be 50 ± 3% and 82 ± 4%, respectively, in 24 h. However, the MX solution released 99 ± 4% in 2 h. MX-BSop-Ig4 exhibited a significantly slower drug release level than MX-Ig. In this case, MX needed to cross the bilosome bilayer and then the polymer matrix. Therefore, slower drug release was achieved. It also exhibited a dual release pattern: an initial fast release and a later slow release. MX-Ig exhibited less release of MX than the MX solution due to higher viscosity. Various kinetic models were calculated to determine the release mechanism. The R^2^ for each kinetic model was calculated, i.e., R^2^ = 0.7525 for zero-order, R^2^ = 0.9053 for first-order, R^2^ = 0.8887 for Higuchi, R^2^ = 0.9466 for the Korsmeyer–Peppas model, and R^2^ = 0.8625 for the Hixon–Crowell model. Based on the maximum R^2^ value, the Korsmeyer–Peppas model was considered the best-fit kinetic release model. The exponent n = 0.58 indicated anomalous transport, i.e., the release of the drug from MX-BSop-Ig by a diffusion mechanism.

#### 2.4.4. Bio-Adhesive Study

The bio-adhesive power of MX-BSop-Ig4 was analyzed with a physical balance technique and was found to be 967.9 dyne/cm^2^. The bio-adhesive power of MX-BSop-Ig4 was significantly more than the shear force tear film (150 dyne/cm^2^) due to the use of chitosan and sodium alginate as gelling agents. The high bio-adhesive power revealed that MX-BSop-Ig4 increased the corneal residence time and would not be eliminated easily by blinking and tear fluid turnover. Similar types of findings have been shown for both acetazolamide-loaded nano-emulsion in situ gel [47] and brinzolamide-loaded noisome in situ gel [48]. 

#### 2.4.5. Permeation Study

A comparative study was performed with formulations of MX-BSop-Ig4, MX-Ig, and pure MX to evaluate the difference in the amounts of MX permeated across the cornea. The order of permeation was found to be MX-BSop-Ig4 > MX-Ig > pure MX. MX permeated through the goat cornea was 63.2% from MX-BSop-Ig4, 50.7% from MX-Ig, and 22.9% from the pure MX solution. MX-BSop-Ig4, MX-Ig, and pure MX showed flux values of 159.7 µg/cm^2^·h, 128.3 µg/cm^2^. H, and 57.9 µg/cm^2^·h, respectively. As a result, MX-BSop-Ig4 performed 1.2-fold better than MX-Ig and 2.8-fold better than the pure MX solution. The high flux of MX-BSop-Ig4 compared to MX-Ig was due to the presence of the lipid, surfactant, SDC, nanovesicle, and bio-adhesive gelling agents. MX-BSop-Ig4 increased the corneal permeation because the BS component could interact with the tear film to increase the ocular residence time and avoid the loss of MX by tear fluid turnover. The high permeation of MX-BSop-Ig4 may be due to the formation of a film over the corneal epithelium and deliberately released [49]. In addition, the high permeation was due to the nanovesicle of MX, which could be internalized in the corneal cell by receptor-mediated endocytosis. The bio-adhesive and penetration-enhancing natures of CH and SA helped the MX to achieve high permeation [50,51].

#### 2.4.6. Corneal Hydration Study

A corneal hydration study was performed to evaluate the tolerance power of the cornea after treatment with MX-BSop-Ig4. The resulting corneal hydration was 77.4%, which was below the normal range (76–80%) [52]. It indicated that there was no damage to the cornea during the experimental study. Therefore, we could say the prepared formulation was safe for ocular delivery. A hydration level greater than 83% indicates damage to the cornea [53].

#### 2.4.7. Histopathological Study

**Figure 9:**
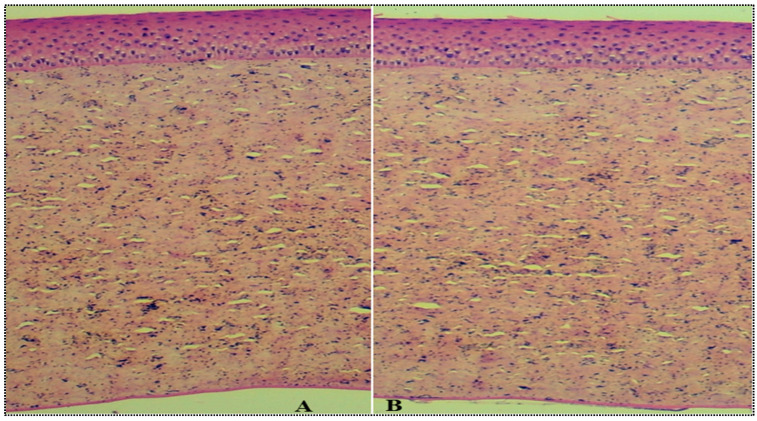
Histopathology images of (**A**) moxifloxacin-loaded bilosome in situ gel (MX-BSop-Ig4) and (**B**) normal-saline-treated cornea.

Figure 9A,B shows histopathological images treated with MX-BSop-Ig4 and 0.9% normal saline. They give an idea of the internal damage to the cornea after treatment with the samples. The excised goat cornea treated with MX-BSop-Ig4 (Figure 9A) showed no damage or changes in its normal structure. The cell structure looked similar to the 0.9% NaCl-treated cornea (Figure 9B). Therefore, from the results, we could say that the prepared formulation, as well as the excipients used to prepare MX-BSop-Ig4, did not produce any toxicity or irritation to the cornea. It revealed that the formulation was safe for ocular administration to treat bacterial infections.

#### 2.4.8. HET-CAM Study

**Figure 10:**
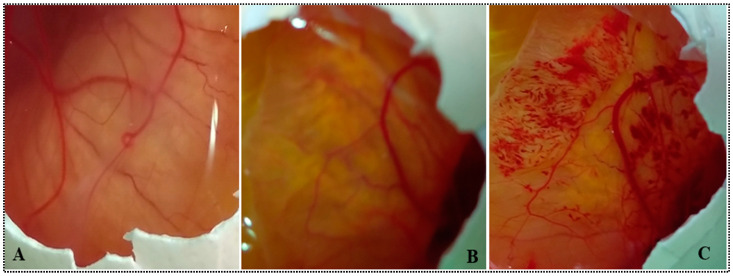
HET-CAM images of (**A**) normal saline, (**B**) moxifloxacin-loaded bilosome in situ gel (MX-BSop-Ig4), and (**C**) positive-control-treated cornea.

Figure 10 represents the HET-CAM images of samples treated with 0.9% NaCl (A), MX-BSop-Ig4 (B), and 0.1N NaOH (C). MX-BSop-Ig4-treated eggs exhibited a score of less than 0.9, indicating no damage to the blood capillaries and veins of CAM. The score revealed that the formulation did not cause any irritation and was found to be safe for ocular administration. However, the positive-control (0.1 N NaOH)-treated eggs exhibited scores of 18.8 (strong irritation), which meant severe damage to CAM. The negative-control (0.9% NaCl)-treated CAM was also found to be a nonirritant. The score was found to be within the standard limit of nonirritant. The findings of the study indicated that the ingredients used to formulate MX-BS-Ig were safe for ocular administration. This result agreed with previously reported research, i.e., besifloxacin and acyclovir nano-emulsion, and did not show any damage to the CAM with a score closer to zero [15,54].

#### 2.4.9. Isotonicity Test

**Figure 11:**
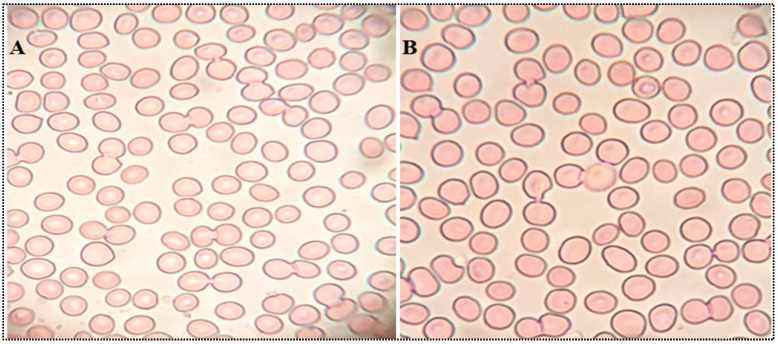
Isotonicity images of (**A**) normal saline and (**B**) moxifloxacin-loaded bilosome in situ gel (MX-BSop-Ig4).

Figure 11 depicts the isotonicity results of MX-BSop-Ig4 and the control (0.9% NaCl) group treated with the blood sample. Both MX-BSop-Ig4 and the control (0.9% NaCl) did not show any changes in the shape of RBCs (shrinking and swelling) when treated with blood (Figure 11A,B). It could be concluded that MX-BSop-Ig4 was isotonic and safe for ocular administration. The results supported the findings of the HET-CAM test.

#### 2.4.10. Antimicrobial Activity Evaluation

**Figure 12:**
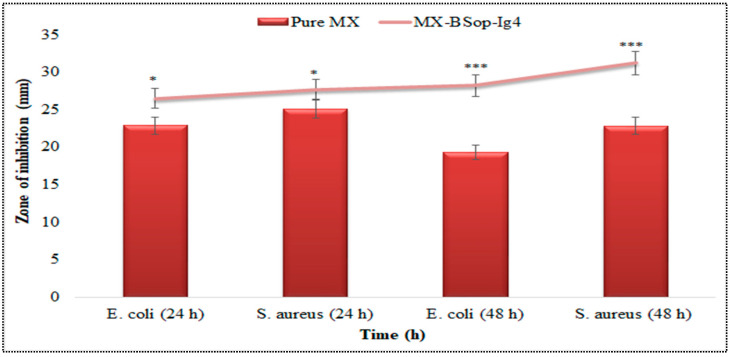
Antibacterial activity of MX-BSop-Ig4 and pure MX solution treated against *E. coli* and *S. aureus.* The study was performed in triplicate, and data are shown as mean ± SD. * indicates significant and *** indicates highly significant to pure MX.

The antibacterial activity of MX-BSop-Ig4 and a pure MX solution was analyzed with zone of inhibition evaluation. The test was performed using the cup-plate method, and the results are depicted in Figure 12. The pure MX (0.5% *w*/*v*) solution showed a ZOI of 22.1 ± 1.3 mm against E. coli (Gram-negative) and 26.5 ± 1.7 mm against *S. aureus* (Gram-positive) at 24 h. The ZOI was decreased at 48 h; it showed 19.3 ± 1.3 mm and 22.8 ± 1.7 mm against *E. coli* and *S. aureus*, respectively. The decrease in activity was found to be more pronounced after 48 h. However, MX-BSop-Ig4 exhibited significantly (*p* < 0.001) higher antibacterial activity than the pure MX solution at both time points. At 24 h, the ZOI was found to be 26.5 ± 1.8 mm against *E. coli* and 27.7 ± 2.2 mm against *S. aureus*. The activity slightly increased at 48 h. It showed a ZOI of 28.2 ± 1.8 mm against E. coli and 31.2 ± 2.2 mm against *S. aureus*. The difference was found to be highly significant at both times in comparison to the pure MX solution. The high antibacterial activity of MX-BSop-Ig4 was due to the nano-sized of BSs [55], which provides a large surface area for the diffusion of MX. The presence of CH and bile salts in the formulation also promoted the antibacterial properties [56,57]. The presence of bile salts and surfactants in the formulation led to the higher solubility of MX and enhanced the therapeutic efficacy. The presence of CH has been reported to have antibacterial activity [58]. It acts by binding to a negatively charged bacterial cell wall. It also helps to alter membrane permeability and inhibits DNA replication, leading to cell death [59]. A similar type of finding was reported in MX-loaded niosomes in-situ gel. It showed higher in vitro activity than MX niosomes against *S. aureus* [32].

#### 2.4.11. Minimum Inhibitory Concentration (MIC)

The MIC values of the pure MX and MX-BSop-Ig4 were determined against both organisms (*E. coli* and *S. aureus).* A significant (*p* < 0.05) difference in the results was found. MX-BSop-Ig4 showed a two-fold lower MIC than pure MX against *E. coli* and a four-fold lesser MIC against *S. aureus.* The pure MX showed an MIC of 0.8 µg/mL against both the organism and MX-BSop-Ig4, which showed 0.4 µg/mL and 0.2 µg/mL, respectively.

## 3. Conclusions

In this work, the MX-loaded BS was successfully prepared and optimized using Box–Behnken design software. The prepared formulation exhibited a nanosized range, high entrapment efficiency, and low PDI. It was further converted into an in-situ gel using chitosan and sodium alginate. The optimized formulation, MX-BSop-Ig4, quickly converted into a gel system on contact with simulated tear fluid and was found to be stable for more than 24 h. MX-BSop-Ig4 showed optimum viscosity, bio-adhesion, and sustained release profile up to 24 h. It showed high flux compared to MX-Ig and a pure MX solution and did not exhibit any damage to the cornea. It did not show any irritation potential to the cornea, confirmed by an in vitro HET-CAM test. It also exhibited significantly higher (*p <* 0.05) antimicrobial activity than pure MX against *S. aureus* and *E. coli*. As per the findings, it can be concluded that a moxifloxacin-incorporated in situ gel system is a potential delivery system for improving the management of bacterial infection.

## 4. Material and Experimental 

### 4.1. Materials

Moxifloxacin was procured from Zydus Cadila Ltd., (Ahmedabad, India). Sodium alginate (SA), cholesterol (CHO), Span 60, and sodium deoxycholate (SDC) were obtained from SD Fine Chemical (Mumbai, India). Chitosan (CH), acetonitrile, chloroform, methanol, and dialysis bags (MWCO 12 kDa) were procured from Sigma Aldrich (Bengaluru, India). Fluid thioglycolate and soya bean casein digest media were obtained from Hi-media (Mumbai, India). All the chemicals used for this project were analytical-grade. 

### 4.2. Experimental

#### 4.2.1. Optimization

The MX-loaded bilosomes (MX-BS) were optimized with a Box–Behnken design using 3 factors at 3 levels [31,60]. Based on the preliminary study, bile salt (SDC, A), an edge activator (Cremophor EL, B), and a surfactant (Span 60, C) were selected as the variables. Their effects were observed on the hydrodynamic diameter (nm as Y_1_), entrapment efficiency (% as Y_2_), and PDI (Y_3_), as shown in Table 3. The selection of the optimized composition was performed using the point prediction method. The desirability value of each response was also observed to check the model validity. The data (polynomial equations and 3D response plots) of all the responses were used to find the relationship between the independent variables and their responses. The experimental models, such as linear, second-order (2F1), and quadratic models, were analyzed to check the effects of the independent variables on the dependent variables. The regression coefficients of all the models, as well as an analysis of variance (ANOVA) of the best-fit model, were calculated.

#### 4.2.2. Development of MX-BS

The MX-BS was prepared with a slightly modified thin-film hydration method, reported by Wilkhu et al. (2014) [19], using bile salt (SDC), Cremophor EL, cholesterol (CHO, fixed concentration, 15%) and a surfactant (Span 60). The detailed composition of MX-BS is shown in Table 1. The ingredients were dissolved in an organic solvent (chloroform: methanol, 1:1). The mixture was transferred into a round-bottom flask and rotated in a rotary evaporator (BUCHI, Flawil, Switzerland) at 50 °C to evaporate the organic solvent under reduced pressure. A thin layer was formed on the surface of the round-bottom flask and was kept overnight in a desiccator to complete the removal of moisture. The film was hydrated for 1 h by taking a weighed quantity of SDC aqueous solution (10 mL) in a rotatory evaporator at 40 °C. Finally, the prepared MX-BS dispersion was further ultrasonicated for 1 min/cycle at 30 s intervals to reduce the size.

### 4.3. Characterization 

#### 4.3.1. Vesicle Characterization

The hydrodynamic diameter, PDI, and zeta potential of MX-BS were analyzed with a size analyzer (Malvern ZS 100, Malvern, UK). The MX-BS formulations (0.1 mL) were diluted with double-distilled water (100 times), and the hydrodynamic diameter and PDI were measured. The surface charge on the vesicle was analyzed using the same samples by placing them in a special electrode cuvette. 

#### 4.3.2. Encapsulation Efficiency

The encapsulation efficiency was determined with an ultracentrifugation method [18]. The percentage of the EE was calculated with following equation:

(4) $$\%\;\mathrm{EE}=\frac{\mathrm{Total}\;\mathrm{MX}-\mathrm{free}\;\mathrm{MX}}{\mathrm{Total}\;\mathrm{MX}}\times 100$$

#### 4.3.3. Drug Loading 

The drug loading of the optimized MX-BS was determined with an ultracentrifugation method, and it was calculated with the given equation:

(5) $$\%\;\mathrm{Drug}\;\mathrm{loading}=\frac{\mathrm{Total}\;\mathrm{MX}-\mathrm{free}\;\mathrm{MX}}{\mathrm{Total}\;\mathrm{weight}\;\mathrm{of}\;\mathrm{BS}}\times 100$$

#### 4.3.4. Development of MX-BS In Situ Gel (MX-BS-Ig)

The point-prediction-optimized formulation, MX-BSop, was further transformed into an in-situ gel system using biocompatible natural polymers (CH and SA), as shown in Table 2. A fixed concentration of the CH solution was prepared in 0.5% aqueous glacial acetic acid. Then, different concentrations of SA were added to the CH solution with continuous stirring for complete solubilization. The MX-BSop was added to the CH and SA solutions to prepare the final concentration of MX (0.5%). Finally, 0.01% methylparaben was added as a preservative and mixed uniformly for further evaluation.

### 4.4. MX-BS-Ig Characterization

The prepared MX-BSop-Ig formulations were assessed for gelling capacity, clarity, pH, and viscosity. The gelling strength of MX-BSop-Ig was determined by placing a 100 µL sample into 2 mL simulated tear fluid (STF, pH 7.4). The time taken to form the gel was noted. The clarity of the sample was checked visually under a black-and-white background by dissolving the formed gel. The pH of the MX-BSop-Ig formulations was analyzed with a digital pH meter (Hanna, Europe). The transmittance of the MX-BS-Ig was inspected with a UV-visible spectrophotometer at 480 nm and compared with STF as a blank. The viscosity was analyzed with a viscometer (Fungi Lab, Barcelona, Spain) in sol (pH 5.5) and gel states (7.4). The drug content was also evaluated by taking a sample (1 mL) and dissolving it in distilled water. The sample was centrifuged at 5000 rpm for 30 min, and the absorbance was analyzed with a UV-spectrophotometer (Genesys 10S UV-Vis, Thermofisher, Waltham, MA, USA) at 290 nm [61].

#### 4.4.1. Differential Scanning Calorimetry (DSC)

DSC thermograms of MX, CHO, Span 60, MX-BSop, SA, CH, and optimized MX-BSop-Ig were recorded using a DSC instrument (Mettler, Toledo, ME, USA). Approximately 4 mg of each sample weight was placed into an aluminum pan and pressed using hydraulic pressure. Then, the pan was placed into the instrument and scanned between 25–400 °C under a flow of nitrogen.

#### 4.4.2. Fourier-Transform Infrared Spectroscopy (FTIR)

IR spectra of MX, CHO, Span 60, MX-BSop, SA, CH, and MX-BSop-Ig4 were recorded to evaluate the drug–polymer interaction study. The sample was placed into a sample holder and scanned between 4000–500 cm^−1^. The spectra were recorded, and changes in the peak height and peak position were evaluated to assess the interactions between pure MX and the carriers.

#### 4.4.3. In Vitro Dissolution Study

The dissolution study of the optimized MX-BSop-Ig4, moxifloxacin in situ gel (MX-Ig), and pure MX was performed using a dialysis membrane [62]. A presoaked dialysis membrane was taken, and the tested samples (1 mL) were filled into test tubes. The dialysis bag was tied to the mouth of a test tube. The release medium (100 mL STF) was placed into a beaker, and the temperature was maintained at 37 ± 0.5 °C. The test tube was suspended in the release medium, and the dissolution media was stirred at 50 rpm using a thermostat magnetic stirrer. The released aliquots (2 mL) were taken from the beaker at a definite time, and fresh dissolution media was added to maintain a constant volume throughout the study. The absorbance was analyzed with a UV-visible spectrophotometer at 290 nm, and the drug release was calculated using a Microsoft Excel sheet.

#### 4.4.4. Bio-Adhesive Study

The bio-adhesive strength of the optimized MX-BSop-Ig4 was determined using physical balance. A fresh goat cornea was procured from a slaughterhouse and cleaned with normal saline (0.9% NaCl). Then, the cornea was fixed on the opposite side of the pan and placed in a sample holder containing the sample for 5 min. In a second pan, weight was added until the cornea was detached. The bio-adhesiveness of MX-BSop-Ig4 was calculated with the following formula:

(6) $$\mathrm{Bioadhesiveness}=\frac{\mathrm{Weight}\;\mathrm{in}\;\mathrm{gram}\;\mathrm{to}\;\mathrm{detach}\;\mathrm{cornea}\;\times \;\mathrm{gravity}\;}{\mathrm{Corneal}\;\mathrm{surface}\;\mathrm{area}\;}$$

#### 4.4.5. Corneal Permeation

This study was conducted using excised goat cornea to check the permeation profiles of the prepared formulations [63]. A fresh cornea was procured from a local slaughterhouse, washed with normal saline, and cut into the specific size of the diffusion cell. Buffer (STF, pH 7.4) was filled in the acceptor compartment of the diffusion cell, and the temperature was maintained at 37 ± 0.5 °C. The excised goat cornea was fixed between the donor and receptor compartments. Samples of MX-BSop-Ig4, MX-Ig, and pure MX solution (1 mL) were added into a donor cell having a surface area of 0.6 cm^2^. At a fixed time, 1 mL of the sample was collected from the acceptor compartment, and the same volume was replaced with fresh STF. The concentration was analyzed with a previously validated HPLC method [64] using acetonitrile and phosphoric acid buffer (30:70% v/v) as the mobile phase. The study was performed at a flow rate of 1 mL/min. The separation was conducted using a C18 column and a UV detector at a wavelength of 254 nm. The amount of the drug permeation flux was calculated using Microsoft Excel.

#### 4.4.6. Corneal Hydration Study

This study was performed to evaluate the corneal tolerance after treatment with the formulations. The excised goat cornea was treated with MX-BSop-Ig4 as per the procedure used for the corneal permeation study. After treatment, the cornea was removed, and the initial weight (wet weight, C1) was noted. The cornea was dried at 60 °C for 72 h, and then the final weight (dry weight, C2) was noted. The corneal hydration was calculated with the given equation:

(7) $$\%\;\mathrm{Corneal}\;\mathrm{hydration}=\frac{C1-C2}{C1}\times 100$$

#### 4.4.7. Histopathological Study

A histopathological examination of the excised goat cornea was performed after treatment with MX-BSop-Ig4 and 0.9% NaCl (control). The cornea was collected and stored in 8% formalin solution. The cornea was dehydrated with alcohol and fixed with paraffin. The cross-section was cut into small, thin slices using a microtome cutter and stained with hematoxylin and eosin dye. The images were captured with a Motic microscope (Fujian, China). 

#### 4.4.8. Chorioallantoic Membrane Study (CAM) 

The HET-CAM study is an alternative method to the Draize test for the analysis of ocular irritation [65]. The study was performed on fertilized eggs. The fertilized eggs were obtained from a local poultry form and incubated for ten days in an incubator at 37 ± 1 °C and 50 ± 2% humidity. On the tenth day, the eggs were removed from the incubator, and the eggshell was removed from the air chamber side. One drop of normal saline solution was added over the inner membrane, and it was carefully removed without damaging the CAM. The eggs were divided into three groups: Group 1 was given 0.9% NaCl as a negative control, Group II was given 0.1 M NaOH as a positive control, and Group III was given MX-BSop-Ig4. The samples (2 drops) were added to the respective CAM groups, and a score was given after visual observation for any changes up to 5 min. The scores were given as nonirritant (0–0.9), slight irritant (5–8.9), moderate irritant (5–8.9), and strong irritant (9–21) [65].

#### 4.4.9. Isotonicity Test

The isotonicity test of MX-BSop-Ig4 was evaluated using goat blood and was compared with normal saline (0.9% NaCl). A drop of MX-BSop-Ig was mixed with blood, and the smear was prepared on a sterilized glass slide. The smear was stained for 5 min with Leishman’s stain. The excess dye was washed with water and air-dried. The slide was observed under a light microscope for any damage to the red blood cells (RBCs).

#### 4.4.10. Antimicrobial Activity Evaluation

The antimicrobial evaluation of pure MX solution and MX-BSop-Ig4 was conducted using the cup-plate method on *Staphylococcus aureus* (*S. aureus*) and *Escherichia coli* (*E. coli*) strains. The medium was prepared with 7.0 g nutrient agar medium, transferred into a conical flask, and dissolved in 250 mL water. The medium was sterilized using an autoclave (Astell, Sidcup, Kent, UK) at 121 °C for 15 min. The medium was removed from the autoclave, cooled, and transferred to a sterilized petri dish with a microbial strain. The plates were kept for solidification, and then a 4 mm well was made with a sterile borer. MX solution and MX-BSop-Ig4 (0.5%) test samples were transferred into the wells. The plate was kept for 2 h and then incubated at 37 °C in an incubator (Binder, Camarillo, California, USA). The zone of inhibition was measured at two timepoints (24 h and 48 h) for each sample against both the test organisms.

#### 4.4.11. Minimum Inhibitory Concentration

The minimum inhibitory concentration of MX was determined using a serial dilution method. A stock solution was prepared and serially diluted to different concentrations. The different concentrations (100 µL) were added to a 96-well plate, and then 20 µL of the inoculum was added. A standard medium was added for the bacterial assay, and the growth of the microorganisms was determined by turbidity. The plates were incubated for 24 h and examined for growth. The MIC was the lowest concentration in the medium that completely inhibited the visible growth.

## Figures and Tables

**Table 1:** Formulation composition of moxifloxacin bilosomes and data of responses.

Formulation Code	Bile Salt (mg)	Edge Activator (mg)	Surfactant (mg)	Hydrodynamic Diameter (nm)	Entrapment Efficiency (%)	Polydispersity Index
	A	B	C	Y_1_	Y_2_	Y_3_
MX-BS1	15	7	45	166	78	0.22
MX-BS2	30	7	45	191	73	0.38
MX-BS3	15	15	45	183	84	0.24
MX-BS4	30	15	45	245	63	0.37
MX-BS5	15	11	30	196	67	0.24
MX-BS6	30	11	30	318	45	0.47
MX-BS7	15	11	60	218	91	0.15
MX-BS8	30	11	60	187	88	0.21
MX-BS9	22.5	7	30	251	53	0.39
MX-BS10	22.5	15	30	245	58	0.41
MX-BS11	22.5	7	60	158	95	0.21
MX-BS12	22.5	15	60	233	86	0.21
MX-BS13 *	22.5	11	45	195	78	0.28
MX-BS14 *	22.5	11	45	195	77	0.29
MX-BS15 *	22.5	11	45	193	76	0.28
MX-BS16 *	22.5	11	45	199	78	0.28
MX-BS17 *	22.5	11	45	192	76	0.28

* Center point (same composition).

**Table 2:** Formulation and characterization parameters of MX-BSop-Ig.

Formulation	CH (%)	SA (%)	Clarity	Optical Transmittance (%)	Gelling Strength		Viscosity		Drug Content (%)
					Sol	Gel	Solution	Gel	
MX-BSop-Ig1	0.35	0.1	Clear	96.7 ± 1.5	−	−	8.4 ± 1.6	28.4 ± 3.8	97.5 ± 2.7
MX-BSop-Ig2	0.35	0.2	Clear	97.3 ± 2.4	−	+	32.5 ± 3.7	133.6 ± 3.1	97.9 ± 2.7
MX-BSop-Ig3	0.35	0.3	Clear	96.3 ± 2.5	−	++	50.4 ± 1.7	166.3 ± 3.1	98.8 ± 1.4
MX-BSop-Ig4	0.35	0.4	Clear	97.0 ± 1.4	−	+++	73.4 ± 5.3	287.5±10.5	99.1 ± 1.2
MX-BSop-Ig5	0.35	0.5	Turbid	80.2 ± 2.3	+	++++	98.5 ± 2.1	314.7 ± 1.3	99.2 ± 2.0

(−) no gel formation and remains liquid; (+) gel forms at 1 min and dissolves after 1 h; (++) gel forms in a few seconds but dissolves within 1 h; (+++) gel forms in a few seconds (2 s) and is stable >24 h; (++++) tough gel forms quickly and is stable for extended time >24 h (turbid medium).

**Table 3:** Independent and dependent variables used to prepare MX-loaded bilosomes (MX-BS).

Independent Variables	Units	Level		
		Low (−)	Medium (0)	High (+)
Bile salt (SDC as A)	(mg)	15	22.5	30
Edge activator (Cremophor EL as B)	(mg)	7	11	15
Surfactant (Span 60 as C)	(mg)	30	45	60
**Dependent variables**				
Hydrodynamic diameter (Y_1_)	nm			
Encapsulation efficiency (Y_2_)	%			
Polydispersity index (PDI) (Y_3_)				

## Data Availability

Not applicable.

## Supplementary Materials

The following supporting information can be downloaded at: https://www.mdpi.com/article/10.3390/gels8070418/s1, Figure S1: IR spectra of moxifloxacin, cholesterol, Span-60, sodium alginate, chitosan, optimized moxifloxacin bilosomes (MX-BSop) and optimized moxifloxacin bilosomes in-situ gel (MX-BSop-Ig4).
