# Supplementary material for: Formulation and Evaluation of Moxifloxacin Loaded Bilosomes In-Situ Gel: Optimization to Antibacterial Evaluation
Source: Gels. 2022 Jul 4;8(7):418. doi: 10.3390/gels8070418 (PMC9323078; doi:10.3390/gels8070418)
Supplement: Supplementary file 1 [file gels-08-00418-s001.zip › gels-1754331-supplementary.pdf]

gels

Article

Formulation and Evaluation of Moxifloxacin Loaded Bilosomes In-Situ Gel: Optimization to Antibacterial Evaluation

Ameeduzzafar Zafar [1,*], Omar Awad Alsaidan [1], Syed Sarim Imam [2], Mohd Yasir [3], Khalid Saad Alharbi [4] and Mohammad Khalid [5]

1 Department of Pharmaceutics, College of Pharmacy, Jouf University, Sakaka 72341, Al-Jouf, Saudi Arabia; osaidan@ju.edu.sa (O.A.A.); kssalharbi@ju.edu.sa (K.S.A.)
2 Department of Pharmaceutics, College of Pharmacy, King Saud University, Riyadh 11451, Saudi Arabia; simam@ksu.edu.sa
3 Department of Pharmacy, College of Health Sciences, Arsi University, Asella, 396 Ethiopia; mohdyasir@arsiun.edu.et (M.Y.)
4 Department of Pharmacology, College of Pharmacy, Jouf University, Sakaka, 72341, Al-Jouf, Saudi Arabia. kssalharbi@ju.edu.sa (K.S.A.)
5 Department of Pharmacognosy, College of Pharmacy, Prince Sattam Bin Abdulaziz University, Al-Kharj 11942, Saudi Arabia; m.khalid@psau.edu.sa (M.K.)
* Correspondence: azafar@ju.edu.sa

Supplementary Materials

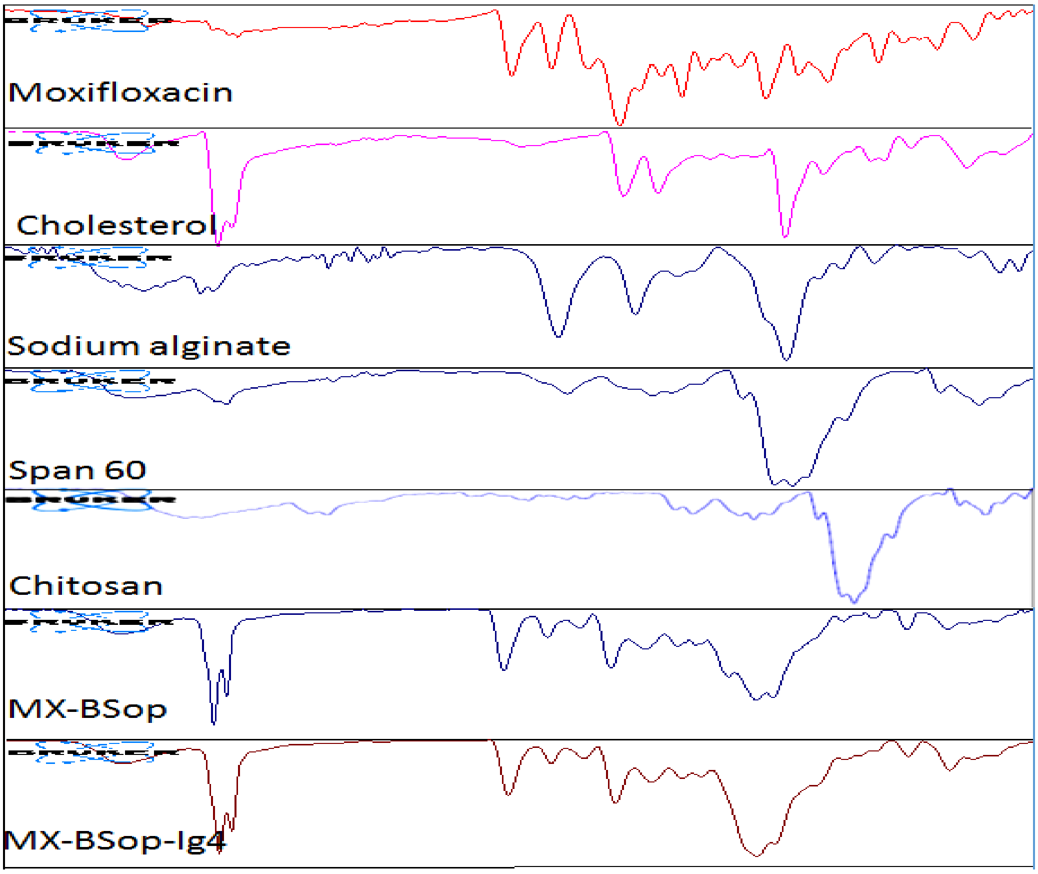

Figure S1. IR spectra of moxifloxacin, cholesterol, Span-60, sodium alginate, chitosan, optimized moxifloxacin bilosomes (MX-BSop) and optimized moxifloxacin bilosomes in-situ gel (MX-BSop-Ig4).
